# The Role of Multimodality Imaging in Patients with Congenital Heart Disease and Infective Endocarditis

**DOI:** 10.3390/diagnostics13243638

**Published:** 2023-12-11

**Authors:** Sara Moscatelli, Isabella Leo, Francesco Bianco, Elena Surkova, Théo Pezel, Natasha Alexandra Donald, Elizabeth Katherine Anna Triumbari, Pier Paolo Bassareo, Akshyaya Pradhan, Andrea Cimini, Marco Alfonso Perrone

**Affiliations:** 1 Inherited Cardiovascular Diseases, Great Ormond Street Hospital, Children NHS Foundation Trust, London WC1N 3JH, UK; sara.moscatelli@gosh.nhs.uk (S.M.); natasha.donald@gosh.nhs.uk (N.A.D.); 2 Institute of Cardiovascular Sciences, University College London, London WC1E 6BT, UK; 3 Department of Experimental and Clinical Medicine, Magna Graecia University, 88100 Catanzaro, Italy; i.leo@rbht.nhs.uk; 4 CMR Unit, Cardiology Department, Royal Brompton and Harefield Hospitals, Guys’ and St Thomas’ NHS Trust, London SW3 5NP, UK; 5 Cardiovascular Sciences Department, AOU “Ospedali Riuniti”, 60126 Ancona, Italy; francesco.bianco@ospedaliriuniti.marche.it; 6 Department of Echocardiography, Royal Brompton and Harefield Hospitals, Guy’s and St Thomas’ NHS Foundation Trust, London SW3 5NP, UK; elena.surkova.md@gmail.com; 7 Département de Cardiologie, Université Paris-Cité, Hôpital Universitaire de Lariboisière, Assistance Publique des Hôpitaux de Paris (APHP), Inserm UMRS 942, 75010 Paris, France; theo.pezelccf@gmail.com; 8 Department of Biomedicine and Prevention, University of Rome Tor Vergata, 00133 Rome, Italy; 9 School of Medicine, University College of Dublin, Mater Misericordiae University Hospital, Children’s Health Ireland Crumlin, D07 R2WY Dublin, Ireland; piercard@inwind.it; 10 Department of Cardiology, King George’s Medical University, Lucknow 226003, India; akshyaya33@gmail.com; 11 Nuclear Medicine Unit, St. Salvatore Hospital, 67100 L’Aquila, Italy; 12 Division of Cardiology and CardioLab, Department of Clinical Sciences and Translational Medicine, University of Rome Tor Vergata, 00133 Rome, Italy; 13 Clinical Pathways and Epidemiology Unit, Bambino Gesù Children’s Hospital IRCCS, 00165 Rome, Italy

**Keywords:** infective endocarditis, congenital heart disease, multimodality imaging, cardiac magnetic resonance imaging, nuclear imaging, echocardiography, cardiac computed tomography

## Abstract

Infective endocarditis (IE) represents an important medical challenge, particularly in patients with congenital heart diseases (CHD). Its early and accurate diagnosis is crucial for effective management to improve patient outcomes. Multimodality imaging is emerging as a powerful tool in the diagnosis and management of IE in CHD patients, offering a comprehensive and integrated approach that enhances diagnostic accuracy and guides therapeutic strategies. This review illustrates the utilities of each single multimodality imaging, including transthoracic and transoesophageal echocardiography, cardiac computed tomography (CCT), cardiovascular magnetic resonance imaging (CMR), and nuclear imaging modalities, in the diagnosis of IE in CHD patients. These imaging techniques provide crucial information about valvular and intracardiac structures, vegetation size and location, abscess formation, and associated complications, helping clinicians make timely and informed decisions. However, each one does have limitations that influence its applicability.

## 1. Introduction

Patients with congenital heart disease are well documented to be at increased risk of infective endocarditis (IE) when compared to the general population regardless of whether they remain unrepaired or have undergone surgical intervention [1]. The aetiology of IE in CHD patients can be ascribed to an increased likelihood of microbial attachment to prosthetic material such as valves, grafts, or central lines; and to damaged endocardium caused by non-laminar blood flow through altered intracardiac anatomy [2].

The potential morbidity and mortality associated with IE is significant, and its treatment requires extended hospitalisation, with around half of the patients undergoing surgical intervention [3]. Timely and accurate diagnosis is therefore vital. However, this is often challenging due to the heterogeneity of the patients involved and their frequently non-classical presentation.

The 2015 AHA guidelines on the diagnosis and management of IE advise patients undergo prompt echocardiography and have blood cultures taken from three separate sites. Based on the Modified Duke Criteria, a diagnosis of IE can then be confirmed, rejected, or considered possible [4]. However, whilst these diagnostic criteria are well evidenced, there remain many cases where IE is culture negative or where echocardiography, either transthoracic (TTE) or transoesophageal (TOE), is equivocal, particularly in patients with prosthetic valves or implantable devices. Furthermore, whilst TOE is superior in diagnostic yield to TTE, particularly in the assessment of valvar vegetations [5], its use is often limited by the availability of resources or the risk to the patient of this more invasive procedure.

There is therefore clear value in the use of adjunctive imaging modalities in cases where diagnostic uncertainty persists [6,7]. Signs of infective endocarditis assessed on imaging include thickening of vessel or prosthetic walls, abscess, pseudoaneurysm, dehiscence of surgical margins, vegetations, and evidence of peripheral seeding of infective emboli or a peripheral source of the intracardiac infection. In addition to initial diagnosis, multimodality imaging can aid prognostication, decisions around surgical intervention, and assessment of response to therapy in patients with IE. In particular, the use of whole-body or extended cardiovascular and neurovascular imaging can be used to detect more distant lesions or provide alternative diagnoses [8]. The updated 2023 ESC guidelines therefore recommend clinicians with expertise in multimodality imaging must be incorporated into the ‘Endocarditis Team’, and its use should be strongly encouraged, though more specific guidance on patient selection and timing is not yet provided [9].

This review will collate the evidence for the role of multiple imaging modalities in diagnosing patients with infective endocarditis, including transthoracic and transoesophageal echocardiography, computed tomography (CT), cardiac magnetic resonance imaging (MRI), and nuclear imaging.

## 2. The Role of Echocardiography

### 2.1. Introduction

Echocardiography is the key imaging modality essential not only in diagnosing IE, but also in guiding the risk stratification and management strategy. Both European and American Guidelines recommend TTE as the first-line imaging modality in patients with suspected IE (class I, Level of Evidence (LoE) B). TOE is useful for further assessment of patients with high clinical suspicion but negative or inconclusive TTE, or in the presence of prosthetic material in the heart (Class I, LoE B) [9,10]. Guidelines also recommend a repeat echocardiographic examination in cases where an initial study does not show any evidence of IE but there is a high clinical suspicion [9]. A repeat examination is also recommended in the setting of suspected new complications (for example, embolism, abscess, or heart failure), and at the end of the antibiotic therapy to assess the response to treatment and sequalae of the IE episode. Staphylococcus Aureus bacteriemia is the most frequent cause of IE and a thorough TTE or TOE examination should be considered in this setting even in the absence of specific IE symptoms [9,10].

### 2.2. Diagnostic Criteria and Suggested Protocol

Originally proposed in 1994 and then modified in 2000, the Duke Criteria provide a diagnostic classification for IE that does not account for the presence of CHD [11,12]. Either a combination of two major criteria, one major and three minor, or five minor criteria is required for a definite IE diagnosis [12,13]. Echocardiographic findings are currently included among major criteria, with three pathognomonic features of IE:

1. Vegetation: echo-dense mass, mobile or non-mobile, usually attached to valvular or endocardial structures or to a prosthetic material [13,14]. The presence of endocardial damage or a differential pressure between two chambers (either between atrium and ventricle, or left and right chambers in case of intracardiac shunt) can facilitate their formation. Valve vegetations tend to be found on the low-pressure surface. They are, therefore, commonly facing the atria if located on the atrioventricular valves or located on the ventricular side if involving the aortic or pulmonary valves [14]. The density of these masses is often comparable to the myocardium but can be increased in case of older lesions, often presenting with areas of calcification.
2. Abscess: echo-dense or echo-lucent lesion, often non-homogeneous, characterised by the absence of colour Doppler signal and often located in the proximity of the valves. Abscesses are more frequently associated with aortic valve IE, where they are usually located within the mitro-aortic junction or around the aortic root [13].
3. New dehiscence of a prosthetic valve: IE should be suspected in case of new onset of paravalvular regurgitation. A rocking motion of the prosthesis may also be present.

Structural valve disruption, leaflet prolapse, valve perforation (particularly of the mitral valve in cases of aortic IE), pseudoaneurysm, valvular aneurysm, or the presence of a fistula are other common features of IE [13]. Beyond acquisition of the standard echocardiographic views, a careful evaluation of the hemodynamic consequences of any observed valvular abnormalities alongside quantification of cardiac output and estimation of pulmonary arterial and ventricular filling pressures is strongly recommended to guide management [13].

TOE is more sensitive than TTE for the detection of vegetations (85–90% vs. an estimated 75%, respectively) and abscesses (around 90% vs. around 50%, respectively) and may be particularly useful in the assessment of prosthetic valves and pacemaker leads [13,15,16]. A specificity of about 90% has been reported for both modalities [14]. Detailed, real-time, three-dimensional (3D) echocardiography assessment may be particularly useful in this context, increasing diagnostic accuracy and enabling better surgical planning. A study including 60 patients with IE demonstrated good feasibility of 3D TOE in identifying vegetations, along with better prognostic value when compared to 2D TOE in terms of embolic risk prediction [17]. Colour Doppler imaging should be added to 3D data acquisition to better visualise regurgitant jet(s) and to distinguish dropout artifacts from leaflet perforation or periprosthetic leak [18].

Echocardiography also helps to identify patients at higher risk of embolic complications, who may benefit from prompt surgical management. For example, vegetations larger than 10 mm in size have been associated with increased risk of embolic events. The risk is even greater if the lesions are highly mobile or larger than 15 mm [19,20]. In addition, vegetations located on the mitral valve, particularly on the anterior leaflet, carry a higher risk of embolization [13].

### 2.3. Echocardiography in IE and CHD

**Figure 1:**
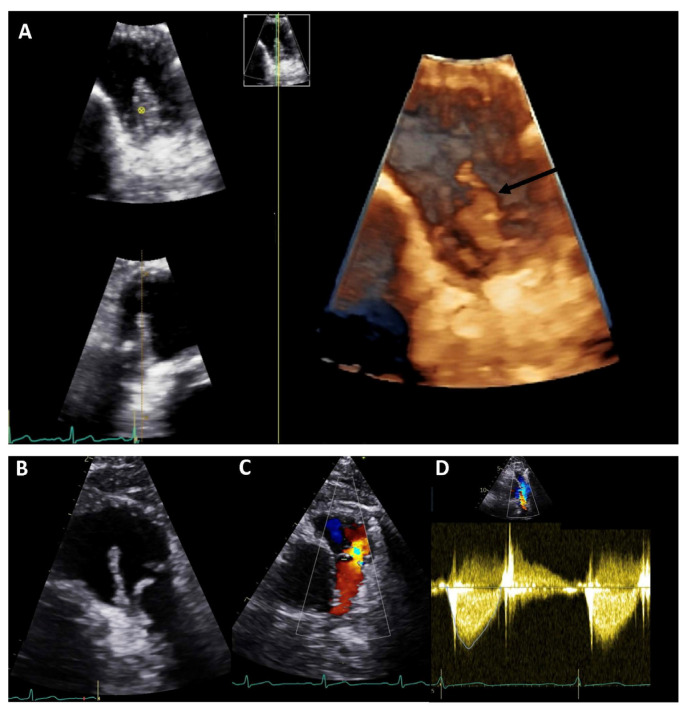
Transthoracic echocardiography of an adult patient with previous Ross procedure for bicuspid aortic valve using a pulmonary homograft. There are large mobile vegetations (max ~18 × 7 mm, (**A**,**B**), black arrows) on the pulmonary homograft. Severe pulmonary regurgitation (regurgitant jet originating in the main pulmonary artery, vena contracta 8.5 mm, (**C**)); no significant stenosis (mean gradient 13 mmHg, peak gradient 26 mmHg, (**D**)). The findings are consistent with infective endocarditis. Images courtesy of Ms Joane Daradar—Echocardiography Department, Royal Brompton and Harefield Hospitals, London.

The contemporary CHD population has changed recently due to therapeutical and surgical advances. Most of the evidence available in the literature comes from since-centre retrospective studies and may therefore not reflect the contemporary scenario of IE in CHD patients. The overall incidence of IE is, in fact, higher in ACHD (1.33 cases/1000 person-years) and in children with CHD (0.41 cases/1000 per year) compared to general the population (~3–5 cases/100,000 py) [21,22]. The recurrence rate has been estimated at 20% [23]. IE is most commonly observed in complex lesions, ventricular septal defects (VSDs), particularly when not repaired, and in carriers of prosthetic materials. Notably, this is particularly true for prosthetic valve and conduit for which the risk of IE is higher in the first years after implantation but persists for a lifetime. Non-valvular prosthetic materials (and valve repair) seem to instead carry the risk of IE only for the first 6 months after implantation, with important clinical implications [21] (Figure 1).

Right-sided IE is more frequent in CHD patients compared to the general population, as well as mural endocarditis presenting in association with left-to-right shunts [4]. The incidence of pulmonary valve IE is significantly higher in these patients, particularly in the context of repaired Tetralogy of Fallot, pulmonary stenosis or regurgitation, previous pulmonary valve repair or replacement, or right-ventricle to pulmonary artery conduit. Given this condition is very rarely encountered in the general population, it may present a particular diagnostic challenge and remain overlooked. The use of multiple and off-axis unconventional views to better visualise the RVOT as well as the use of TOE to better visualize the more anteriorly positioned PV is therefore strongly recommended in cases of suspected IE involving the PV. As a surrogate indicator of potential IE, a sudden increase in transvalvular gradient on follow-up imaging is a red flag which should prompt the clinician to strongly consider this diagnosis [24]. A systematic review of the literature, conducted in 2017, demonstrated higher IE incidence for bovine jugular vein valves (e.g., Melody or Contegra), compared with other types of right ventricle-to-pulmonary artery conduits. These data were confirmed in both surgical and transcatheter implanted valves, leading the authors to conclude it may have been the valve tissue itself which acted as the substrate for future infection [25].

Interestingly, the previously detailed superiority of TOE when compared to TTE in detection of vegetations and abscesses has not been fully established in CHD cohorts. However, results from a Japanese multi-centre collaborative study reported a relatively low detection rate of vegetations on TTE examinations of CHD patients (62%). TTE may therefore be insufficient to pick up IE-related abnormalities in the context of complex anatomy, post-surgical findings, and prosthetic material [26,27]. It is therefore suggested TOE should be used to complement standard TTE to increase the diagnostic accuracy, particularly in this context [27].

### 2.4. Limitations

TTE may not demonstrate any abnormality in an estimated 15% of patients with IE [13]. This may be due to the small size of vegetations, presence of prosthetic material, pre-existent lesions, poor acoustic window, or imaging performed too early in the disease course. Associated cardiac abnormalities and post-repair findings are more common in CHD patients, and examination in a highly specialised centre is key in this context to avoid misdiagnosis. Some lesions such as Lambl’s excrescences, non-infective (marantic) vegetations, Libman-Sacks endocarditis, or Loeffler’s hypereosinophilic endocarditis may also mimic IE and create further diagnostic challenges. The use of 3D echocardiography has an undeniable advantage in these patients; however, its lower temporal and spatial resolution may be a limit in the identification of very small and highly mobile lesions [14]. Finally, the diagnostic value of echocardiography is limited in the assessment of distal structures, for example, prosthetic material placed in pulmonary artery branches, which are difficult to capture within the margins of available echocardiographic windows.

## 3. The Role of Cardiovascular Magnetic Resonance

### 3.1. Introduction

Cardiovascular magnetic resonance (CMR) can play an important role in the diagnosis and management of patients with CHD who are affected by IE. CMR can accurately assess cardiac function, blood flow patterns, and visualise complex cardiac anatomy. It can also detect and monitor complications associated with both CHD and IE, such as valve regurgitation or stenosis, ventricular dysfunction, location and size of vegetations, abscess, or pseudoaneurysm formation [9,28,29,30]. CMR can, indeed, help to differentiate between isolated infection and the presence of myocarditis or pericarditis [9,28,30]. However, the utility of CMR in patients with CHD and IE may be limited for several reasons. For example, introducing foreign material into the bloodstream, such as Gadolinium-Based Contrast Agent (GBCA), can potentially worsen or spread the infection [30]. In addition, mechanical valves or non-MRI compatible devices could be a contraindication to CMR. Its use is also reliant on the availability of this resource along with the specialist expertise to support obtaining and interpreting images [10,31,32]. Therefore, the timing and appropriateness of CMR should be carefully considered for each individual and the specific centre.

### 3.2. Sequences Used during CMR Investigations in Patients with CHD and IE

For patients with both CHD and IE, a CMR protocol must include sequences to evaluate cardiac anatomy, function, and any valvular or structural damage. In addition, it is crucial to include sequences that can assess the presence and severity of pulmonary hypertension and haemodynamic shunts [33]. The CMR protocol for a patient with CHD typically involves dark- and bright-blood single-shot images, balanced steady-state free precession (bSSFP) cine images, flow imaging (either 3-Dimensional (3D) or 4D), and 3D whole-heart images, which are non-contrast-based sequences. If necessary, it will also include contrast agent-based imaging such as angiography and early and late gadolinium enhancement (LGE) [33,34]. In case of suspected IE, the types of CMR sequence used are the same, but these can be modified to focus on the potential complications suspected.

A CMR study usually begins with dark- or bright-blood single-shot imaging in axial, coronal, and sagittal planes of the thorax to plan further sequences and potentially identify any unexpected extra-cardiac findings or complications due to IE, such as suspicion of peripheral embolization [35]. bSSFP cine images can provide information on anatomy, function, and valves and are acquired in long- and short-axis views [33,34,35,36]. Through these sequences, it is possible to assess the presence of vegetations, abscesses, or pseudoaneurysms, even if the lower CMR spatial resolution compared to CT and the lower temporal resolution compared to echocardiography make CMR not the gold standard for these findings. bSSFP can show ventricular dysfunction, damage to valves, and even indirect signs of pulmonary hypertension. Angiography sequences are performed using CBGA and acquired when the agent arrives in the vessel of interest. These can be useful to assess the patency of a conduit involved. Additionally, 3D whole-heart SSFP allows a comprehensive evaluation of intracardiac and extra-cardiac morphology, visualisation of the arterial and venous systems, and measurements of vessel size. Flow across vascular structures can be assessed using two-dimensional (2D) phase-contrast imaging (PCI), which can quantify peak velocities and blood flow volumes, highlighting regurgitation across valves or conduits affected by IE and can suggest the presence of stenosis even if this method is not the gold standard for stenosis quantification. The 4D flow is an extension of the 2D flow, which is currently the most used clinical flow application, and allows comprehensive and accurate assessment of flow in a single acquisition, gaining in time [37].

Finally, LGE sequences, acquired 10 min after the injection of the contrast agent, can detect the presence and extent of any myocardial fibrosis that can be potentially suggestive of a concomitant myocarditis process, even if its actual prevalence is still unknown [5,34]. LGE imaging is the gold standard for assessing myocardial fibrosis, relying on the different washout kinetics of a gadolinium-based contrast agent in healthy and fibrotic myocardium. Early gadolinium sequences can also be acquired immediately after the contrast agent injection to detect the presence of any potential thrombi [33,34,35,36].

### 3.3. Evidence

Despite the numerous benefits of using CMR imaging to assess CHD, endocarditis, and potential cardiovascular complications, there is a shortage of reliable data on its effectiveness within the IE algorithm. Currently, the literature suggests CMR should not replace clinical, microbiological, or echocardiographic evaluation, but rather should be used in conjunction with these methods to improve the accuracy of diagnosis and the presence of complications [9,38,39]. Indeed, the European and American guidelines primarily focus on the role of cerebral magnetic resonance imaging (MRI) and its significance in identifying cerebrovascular emboli. They briefly acknowledge CMR as a new imaging tool that could be incorporated into the diagnostic algorithm [5,9,39,40].

**Figure 2:**
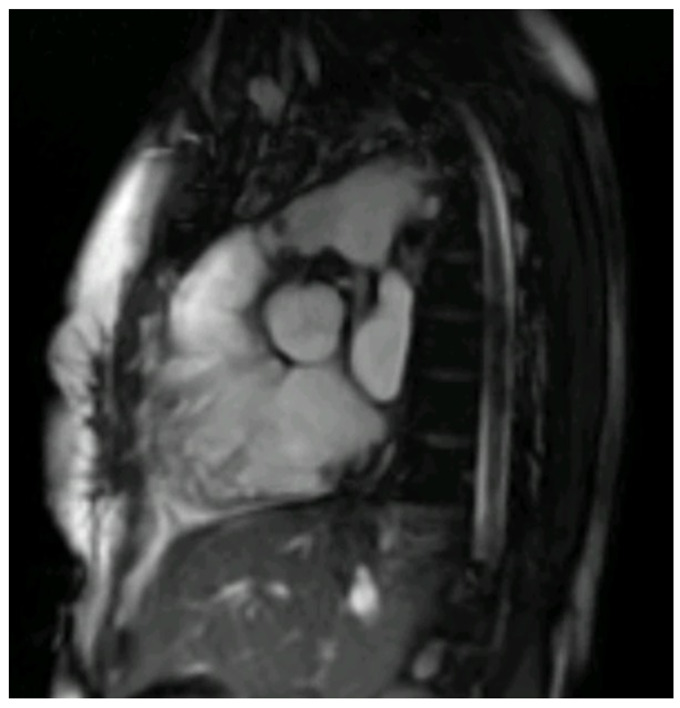
Right ventricle outflow tract bSSFP CMR image with endocarditis vegetation of a thirty-year-old male with Tetralogy of Fallot and a bio pulmonic conduit in the pulmonary position, with positive blood cultures for S. Mitis following dental extraction.

Despite concern over the infection risk associated with contrast agents and MRI’s relative limitations in patients with existing metallic prostheses, CMR may provide superior evaluation of transvalvular flows when utilising 2D PCI flows or 4D flow, which is especially useful in the assessment of valve regurgitation due to perforation or destruction of the cusps or due to paravalvular leakage. Furthermore, in complex haemodynamic scenarios, such as assessment of intracardiac fistula, CMR can be used to more accurately quantify the shunt using 2D PCI flow sequences [36,40]. In terms of accurate diagnosis, CMR with LGE may assist in identifying myocarditis, an important immune-mediated differential of infective endocarditis. Additionally, CMR may be used in favour of other modalities for specific patient situations. For example, it is superior to cardiac computed tomography (CCT) in terms of minimising radiation exposure and may be preferable in patients with renal dysfunction for whom gadolinium-based contrast agents are less nephrotoxic. During COVID-19, to minimise aerosol-generating procedures, the group of S. Bhuta at al. utilised CMR as an alternative to TOE for diagnosing IE. However, CMR demonstrated mixed results in diagnosing valvular vegetations and guiding clinical decision-making, highlighting a need for further prospective controlled trials of CMR versus TOE to better clarify the role of CMR in the IE algorithm [41] (Figure 2).

### 3.4. Limitations and Pitfalls

The application of magnetic fields in CMR makes it theoretically unsuitable for patients with non-MRI conditional devices like pacemakers or ICDs [42,43]. Nonetheless, promising evidence indicates expert centres may safely perform MRI even with non-conditional devices [10,31]. GBCA plays a crucial role in the CMR protocol, but it is associated with adverse reactions, including mild allergic responses and nephrogenic systemic fibrosis (NSF) in individuals with kidney dysfunction (eGFR < 30 mL/min/1.73 m^2^). Repeated administration of the contrast in short time frames may also lead to gadolinium accumulation in the basal ganglia of the brain [44]. Moreover, CMR requires lengthy image acquisition with adherent patient compliance, which may not be feasible in the paediatric population. Typically, it is conducted in awake children aged eight or older, while general anaesthesia or sedation is necessary for younger or non-compliant individuals, which poses additional risks [35,36].

In summary, CMR appears to be a valuable tool in the evaluation and management of patients with both CHD and IE, providing information that can guide therapeutic decisions and improve patient outcomes, but more studies need to be conducted on the topic. However, the use of CMR in these patients must be carefully balanced with the potential risks and contraindications associated with the procedure.

## 4. The Role of Cardiac Computed Tomography

Cardiac computed tomography (CCT) is a complementary tool to standard echocardiography for the diagnosis and management of IE patients; particularly, when TOE is contraindicated, or TTE presents with a poor acoustic window or returns with suboptimal results due to calcifications or the presence of prosthetic valves. In addition, CCT has recently been included in the 2023 ESC Guidelines on IE, into the IE modified diagnostic criteria, underlining the importance of this technique of imaging in this context.

### 4.1. Protocols

Protocols for CCT examinations are comparable to those applied for coronary computed tomography (CT) angiography, either for preparation or acquisition [45].

In particular, CCT with ECG-gated acquisition at 0.60–0.75 mm is generally sufficient to provide an isotropic data set of cardiac structures allowing their assessment in any desired multiplanar orientation. Comparable echocardiographic short- and long-axis or apical views can be reconstructed, thus, permitting images’ comparisons with both techniques [46,47].

Coronary CT angiographies are habitually acquired prospectively, during diastole. Conversely, a multiplanar-multiphase CCT imaging protocol is preferred for evaluation of IE, across retrospectively ECG-gated or wide window ECG-triggered image acquisition [48,49]. When we acquire images at different points of the cardiac cycle, four-dimensional (4D) cine images can be obtained (0–100% reconstruction at 5–10% intervals) in any reconstructed view (i.e., short or long-axis) by combining the 10–20 cardiac phases. The latter is particularly helpful when a qualitative assessment of valve leaflet motion and planimetry is required [46,48].

Regarding contrast media, the injection site, volume, and rate should be tailored to the expected IE cardiac locations. For suspected tricuspid endocarditis, contrast enhancement of the right atrium and ventricle is sufficient. For different sites, venous or arterial and delayed phase injections are indicated and should be selected on a case-by-case basis. In adults, 1.5 mL/kg of contrast media at a rate of 3–4 mL/s is recommended. In neonates and children, the contrast agent injection at a dose of 2 mL/kg (not exceeding 100 mL of total contrast volume administration) is generally enough to obtain good quality images [45,49].

The injection rate is set at 1 mL/s but can be increased to 2 mL/s in patients with larger intracardiac shunts. A saline bolus commonly follows contrast media administration, and reduces the artefact caused by undiluted contrast. In neonates or children, when applying contrast reduction protocols, the total quantity of saline should be reduced proportionally to the contrast to avoid over-dilution [50,51,52] bolus-tracking is mandatory when acquiring ECG-gated CT scans. Accordingly, the region of interest (ROI) in pediatrics can be placed in the left ventricle, at a threshold attenuation of 200 HU. In adults, the ROI is placed in the ascending/descending aorta, at the attenuation threshold of 140/180 HU [45,49,50].

Faure and colleagues reported their experience of a dedicated three-phase acquisition protocol for comprehensive prosthetic heart valve assessment, with a mean dose of 8.3 mSv, that comprises (1) unenhanced imaging of the valve region, (2) dynamic wide window prospective ECG-triggered CT angiography of the heart, and (3) late phase imaging of the entire thorax [51].

In our practice, when intracardiac anatomy is unknown, and there is doubt regarding the possible presence of shunts, baseline CT acquisition is often advisable as well as more careful tracking of the progress of contrast agents. When performing a non-ECG-synchronized CT in pediatric patients, a fixed scan delay is usually sufficient. The starting delay is set at 12 s for peripheral injection and 8 s for central venous injection. Premedication with betablockers or vasodilators is not recommended and not helpful in IE patients unless coronary anatomy also needs to be explored [52].

### 4.2. Role of CCT in CHD Patients Affected by Endocarditis

The diagnosis of IE can sometimes be challenging due to diagnostic limitations of echocardiography, both TOE and TTE, especially when blood cultures are negative, or the pulmonary valve is involved. In fact, the latter can be challenging to demonstrate due to its anterior position within the chest [53].

**Figure 3:**
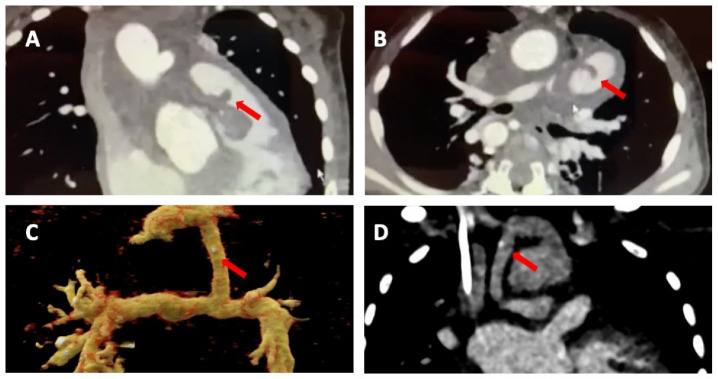
Cardiac computed tomography infective endocarditis visualisation. The images show right-ventricular pulmonary artery (RV-PA) conduit involvement (panels (**A**,**B**)) and systemic to pulmonary shunt involvement with a calcified lesion (panel (**C**,**D**)). The arrows indicate the vegetations.

In this context, Nagiub M. and colleagues presented their single-centre retrospective experience of pediatric patients with CHD and diagnosis of IE in which they described a high proportion of right-ventricular pulmonary artery (RV-PA) conduit involvement (Figure 3) as well as IE of the aortic valve. CCT also demonstrated thromboembolism, pseudoaneurysms, prosthetic valve perforations, and prosthetic valve leaks [6,54]. Furthermore, they discussed more peripheral, multi-site imaging to assess for vegetations and emboli (present in around 20–50% of the cases) [20].

In our own centre’s experience (AOU “Ospedali Riuniti’’, Ancona, Italy), the presence of a ventricular septal defect with their associated vegetations along with systemic to pulmonary shunt localisation present particular challenges (Figure 3). In addition, a recent meta-analysis has shown CCT demonstrated good diagnostic accuracy in terms of assessing valvular and periannular complications of IE. Of note, TOE remained superior for detecting valve leaflet defects [6].

### 4.3. Future Directions

New scanners equipped with photon counting (PC) or dual-source-CT techniques provide a spatial resolution of 0.2 mm (nominally 200 micron) and a higher temporal resolution (nominally below 79 milliseconds). This may play an important role in improving representation of cardiac structures and CT should be considered in selected cases of IE, where echocardiography alone could not provide sufficient information. In particular, PC could be particularly helpful in IE where a pacemaker lead or a ventricular/atrial septal defect inhibits views of a suspected IE lesion. A greater temporal resolution may also improve the characterisation of vegetation involving the valves or other highly mobile heart structures. Higher temporal resolution may also reduce the total scan time permitting faster acquisition in more unwell patients or infants and children, while reducing the length of sedation.

## 5. The Role of Nuclear Molecular Imaging

### 5.1. Introduction

In recent years, there has been growing scientific and clinical data supporting nuclear molecular imaging as an important supplementary diagnostic method for IE and its extra-cardiac complications. In patients with CHD, nuclear molecular imaging overcomes some of the diagnostic limitations presented by other imaging modalities.

Single photon emission tomography/computed tomography (SPECT/CT) with labeled autologous white blood cells (WBC) and Positron Emission Tomography/Computed Tomography (PET/CT) can provide additional diagnostic and prognostic information in this challenging group of patients [54]. The Task Force for the Management of IE of the European Society of Cardiology (ESC), endorsed by the European Association for Cardio-Thoracic Surgery (EACTS) and the European Association of Nuclear Medicine (EANM), proposed to add nuclear medicine imaging results to the modified Duke Criteria [12]. In fact, in selected clinical situations, such application would improve the sensitivity of the currently employed diagnostic algorithms. Therefore, nuclear molecular imaging results will become part of major and minor diagnostic criteria in the forthcoming publication of the 2023 update of the modified Duke Criteria by the International Society for Cardiovascular Infectious Diseases (ISCVID) [55].

### 5.2. SPECT/CT

#### 5.2.1. Protocol

In SPECT/CT, WBC are labeled either with technetium99m-hexamethylpropyleneamine-oxime (^99m^Tc-HMPAO) or Indium-111-oxine (^111^In-oxine). ^99m^Tc-HMPAO is preferred to ^111^In-oxine for better image quality and lower radiation exposure (0.011 mSv/MBq vs. 0.36 mSv/MBq). This modality takes advantage of the chemotactic properties of leucocytes to reveal the presence of infected sites at a whole-body level.

Patient preparation for WBC SPECT/CT requires fasting before blood sample collection. Prior to the imaging protocol, careful blood handling is required for the isolation, radiolabeling, and reinjection of autologous leucocytes (about 50 mL) obtained from the patient’s blood. Strict aseptic conditions are necessary for the labelling procedure. Following, a 24-h-long protocol includes planar early (30–60 min), delayed (2–4 h), and late (20–24 h) acquisition. These should be acquired with a “time corrected for isotope decay” modality, as described in the EANM guideline [56]. Planar acquisitions always include whole-body images and antero-posterior views of the thorax and any other region of interest, to exclude the presence of septic emboli. SPECT/CT images are mandatory and usually acquired 4–6 h and/or 20–24 h after injection. Such modality allows for attenuation correction and concomitant anatomic localization of the functional findings. Signal kinetics between the acquisitions are important features for interpretation: any stable or increased site of uptake (either intensity or size) over time, confirmed on SPECT/CT, is highly suggestive of infection.

#### 5.2.2. Role of SPECT/CT in CHD Patients Affected by IE

In patients with suspected IE, WBC SPECT/CT holds a significantly higher diagnostic accuracy, specificity, and positive predictive value compared to trans-thoracic echocardiography (90%, 88%, and 81% vs. 60%, 42%, and 46%) [57].

It has been shown to help differentiating infectious and sterile echocardiographic lesions on both native and prosthetic valves, reducing by 27% the number of misdiagnosed IE classified in the ‘possible’ category by modified Duke Criteria [57,58,59].

Moreover, it allows for determination of localization and extent of sites of infection, both endocardial and of cardiovascular implantable electronic devices and left-ventricular-assist devices, demonstrating high specificity even early (<3 months) after prosthetic valve or devices insertion [6]. In this latter case, WBC SPECT/CT should represent the imaging modality of choice [6].

Localisation of the sites of concomitant extracardiac infection from septic embolism is also possible on SPECT/CT images, influencing the Duke score and, consequently, the diagnostic certainty and patient management [54].

#### 5.2.3. Limitations

The accumulation of WBC may be influenced by several factors. While very few false positive cases have been reported [60], prior and concomitant antimicrobial treatment significantly impacted the diagnostic properties of labeled WBC SPECT/CT [59].

The small size of vegetations and septic emboli, the type of pathogen involved (e.g., in case of Candida spp., Enterococcus spp., Staphylococcus epidermidis, Enterococcus faecalis), and the vascularization of the infected tissue can also negatively influence results [55].

Embolisms on WBC imaging can appear either as foci of increased uptake, or as cold spots (e.g., in spleen embolism). Therefore, even though these findings are highly suggestive for septic embolism, additional diagnostic imaging tests should be considered.

### 5.3. PET/CT

#### 5.3.1. Protocol

The tracer of choice to detect IE with PET/CT is 2-deoxy-2-[Fluorine-18] fluoro-D-glucose (^18^F-FDG). Chemically, it is a glucose analog with the positron-emitting radionuclide ^18^F in place of the hydroxyl group (-OH) at the 2-C position of the glucose molecule. Once it enters a cell and is phosphorylated to FDG-6-P, it cannot be metabolized further, and it remains trapped in the cell until it becomes dephosphorylated by glucose-6-phosphatase. This enzyme lacks in tumor cells. Therefore, ^18^F-FDG tends to accumulate in neoplastic cells. With some different pharmacodynamic parameters, normal tissues and pathological ones with high glucose demand (e.g., inflammatory cells), also show ^18^F-FDG uptake.

Normal myocardium can show physiological ^18^F-FDG uptake [61,62], reducing the diagnostic specificity of PET/CT for IE [63]. Therefore, the EANM provided some procedural recommendations that suggest, to suppress myocardiocytes’ physiological uptake, patients should be instructed to follow high-fat-enriched diet lacking carbohydrates for 12–24 h prior to the scan, combined with a prolonged fasting period of 12–18 h, with or without the use of intravenous heparin of 50 IU/kg approximately 15 min prior to ^18^F-FDG injection [63]. In addition, strenuous exercise should be avoided for at least 12 h prior to the exam [64].

**Figure 4:**
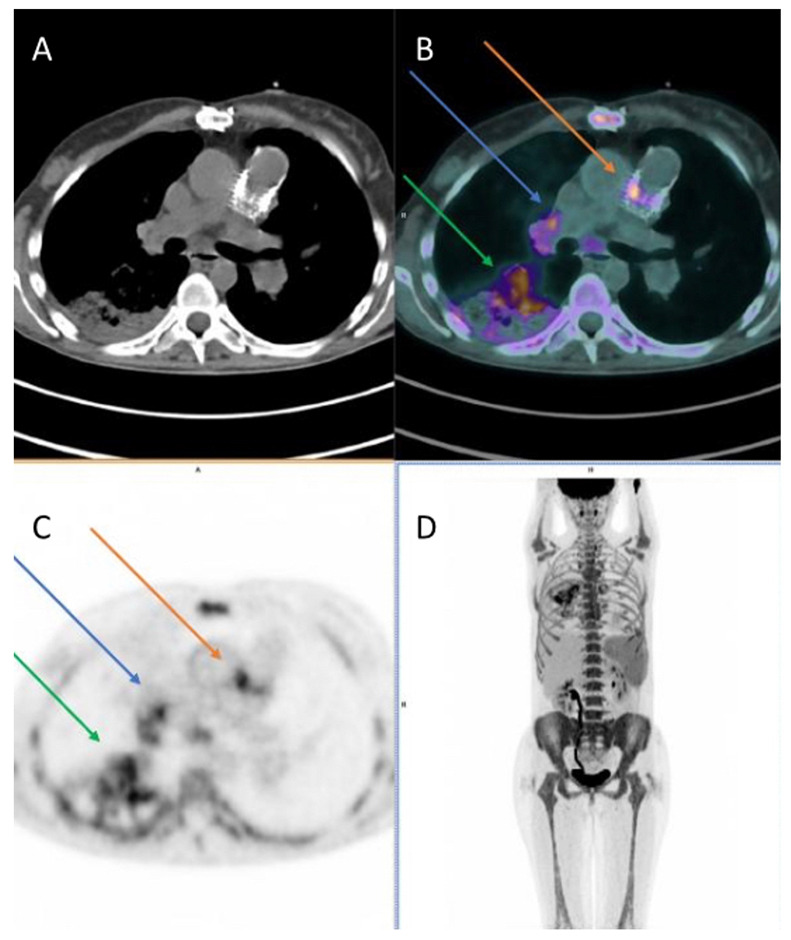
A case of pulmonary valve/valve duct endocarditis of a 38-year-old female patient (surgically treated for Fallot tetralogy in 1988 and 2012), evaluated with ^18^F-fluorodeoxyglucose (^18^F-FDG) positron emission tomography/computed tomography (PET/CT): axial CT, PET/CT, and PET images (**A**–**C**) and maximum intensity projection (MIP) image (**D**) show hypermetabolism in the pulmonary valve prosthesis and in the valve duct (orange arrows), with extension to aortic wall (demonstrating a disease involvement in the aorta as well). Moreover, the images depict multiple reactive hylo-mediastinal lymph-nodes with high uptake of the radiopharmaceutical (blue arrows) and hypermetabolism in an area of consolidation in the right lung (manifestation of pneumonia, green arrows). In addition, image D shows high uptake of ^18^F-FDG in spleen and bone marrow, as a sign of systemic inflammatory response.

Other patient preparation advice and the image acquisition protocol follow the EANM guidelines for oncological ^18^F-FDG imaging [64]. Briefly, the recommended interval between tracer injection (175–350 MBq or 4.7–9.5 mCi in a 70-kg standard adult) and acquisition is 60–90 min. The imaging time is about 2 min/bed position (total of around 15–20 min). Adding gated cardiac PET is optional: it may improve image quality, especially in (prosthetic) valve infective IE assessment, but supporting literature is scarce. PET images have to be visually evaluated for increased ^18^F-FDG uptake, taking into consideration the pattern (focal, linear, diffuse), intensity, and relationship to areas of physiological distribution (Figure 4). Interpretation of nonattenuation-corrected images can avoid attenuation artifacts due to metal implants.

#### 5.3.2. Role of SPECT/CT in CHD Patients Affected by IE

Compared to WBC SPECT/CT, ^18^F-FDG PET/CT has a series of advantages: it has a shorter and easier imaging protocol, a higher photon detection efficiency and spatial resolution, and a lower number of false negatives. These aspects can be practically translated into a faster turnaround time, leading to earlier diagnosis and appropriate interventions [65]. Moreover, it is important to underline, during the past years, ^18^F-FDG PET/CT is gaining a well-established role in the evaluation of disease severity in clinical practice [66].

In patients with CHD, carrying complex anatomy and often large amounts of prosthetic material required for their surgical treatment, use of the modified Duke Criteria is limited for diagnosis. Metal-strut-related artifacts limit the interpretation ability and vegetations’ identification by echocardiography or MRI [67]. Undoubtedly, ^18^F-FDG PET/CT can provide considerable information used for risk stratification and in the decision-making process for treatment strategy planning in selected cases where the diagnosis of IE is uncertain or when involvement of implantable devices is suspected [68]. The added value of PET/CT consists in the possibility of examining the extracardiac portion of leads, device pockets, and the potential ability to differentiate between thrombus and vegetation, especially when compared to transesophageal echocardiography. In association with the Duke Criteria, ^18^F-FDG PET/CT increases sensitivity for a definite diagnosis from 52–70 to 87–97%, reclassifying cases initially deemed as “possible” and providing a more conclusive diagnosis (definite/reject) [14,68,69].

Moreover, when in association to CT angiography (^18^F-FDG PET/CTA), sensitivity and specificity of an infective endocarditis diagnosis in patients with CHD increase and rises to 91%, with 93% positive predictive value and 88% negative predictive value for the detection of prosthetic valves and intracardiac devices infection [70]. Indeed, such hybrid solution combines the highest sensitivity to detect infection with the highest spatial resolution to define structural damage [69].

PET/CT could be considered the first-line nuclear medicine procedure for the detection of extra-cardiac pathology in patients with IE [71,72]. In this setting, PET/CT has demonstrated a significantly higher clinical utility score than WBC SPECT/CT [72]. Except for embolic localization to the brain (which is physiologically ^18^F-FDG-avid), it is in fact able to allow precise localization of septic emboli (even in about 50% of clinically silent patients [68,71,73,74]), determination of the source of infection and concomitant infection, with higher sensitivity and diagnostic accuracy (92% vs. 75%) than WBC-SPECT/CT [74].

^18^F-FDG PET/CT has proven valuable impact on therapeutic decisions, altering treatment plans in more than 30% of cases [71,73]. The greatest consequences especially concern the performance of surgical procedures and the unnecessary device extraction that can be prevented owing to the ^18^F-FDG PET/CT findings.

#### 5.3.3. Limitations

For the chemical intrinsic properties of ^18^F-FDG, PET/CT is less specific than WBC SPECT/CT for the diagnosis of an infection. Indeed, a recent meta-analysis described an overall specificity of 100% (even in the early post-intervention phase) for WBC SPECT/CT, against a pooled 89% for ^18^F-FDG PET/CT. Such values depended on the unspecific ^18^F-FDG accumulation: apart from being physiologically seen in the myocardium [62], it has been described for other diseases and processes such as cardiac tumors, active cardiac sarcoidosis, and even in atheromatous plaques with active inflammation [67]. Therefore, a PET/CT scan was not recommended earlier than 3 months post-surgical treatment, as post-operative inflammation could cause a false-positive interpretation. However, such concern, especially in case of the implantation of a prosthetic valve, is being progressively overcome [75].

Other limitations of ^18^F-FDG PET/CT regard its low sensitivity for native valve endocarditis (that currently does not represent a routine indication for PET), and possible false negatives during antimicrobial treatment. Withdrawal is not routinely recommended, though it can influence PET/CT results. The risk of false negative scans has been reported to be the lowest if the patient was imaged when their CRP is >40 mg/L. On the other hand, whenever clinically safe, steroid treatment should be reduced to the lowest possible dose, if not discontinued at all, in the 24 h preceding the examination.

## 6. Prospects

Regarding promising PET radiopharmaceuticals in this context, interesting results in animal models of IE have been found using new, bacteria-specific, PET radiopharmaceuticals such as Fluorine-18-Fluorosorbitol or Fluorine-18-Fluoromaltohexoase [75]. Moreover, Wardak et al. demonstrated notable results with Fluorine-18-Fluoromaltotriose (a PET radiopharmaceutical that targeted maltodextrin transporter of bacteria) in an animal model, showing bacterial infection of valves with high specificity and sensitivity [75]. Nonetheless, the clinical utility of the aforementioned radiopharmaceuticals should be confirmed in human studies [75].

In the future, PET/MRI platforms may have a role to play in imaging endocarditis in that colocalizing infection/inflammation by PET with tissue characterisation and anatomy by MRI may prove useful [72].

## 7. Discussion

This literature review sought to bring together current theories and evidence around the use of different imaging modalities in the journey of a patient with congenital heart disease with suspected infective endocarditis. There have been previous literature reviews covering the topic of imaging in infective endocarditis; however, the specific population of patients with congenital heart disease has not been a point of focus. These patients constitute a very heterogenous group and are at higher risk of IE than the general population due to the nature of their underlying substrate or their subsequent interventions. However, these features also constitute an additional challenge for the imaging diagnostician in view of the presence of prosthetics which can cause artefact, the presence of unconventional cardiac architecture which can either conceal or mimic the lesions associated with IE, and the prevalence of lesions in areas where traditional echocardiographic views might be limited. It is therefore important to consider the role of adjunctive imaging in this specific patient population and valuable to summarise the information available.

This was structured as a narrative review aiming to summarise current guidance and ideas, providing a point of reference for future focused research questions. It was therefore naturally limited by the inclusion of studies at the authors’ discretion as well as publication bias, as ongoing and unpublished studies were not considered. Furthermore, there were modalities that were beyond the scope of this review, for example intracardiac echocardiography.

While it is clear transthoracic echocardiography is the most widely available modality, with good evidence behind its use as key first line imaging, the additional diagnostic value of cardiac CT over echocardiography and functional imaging with FDG PET/CT over imaging the basic anatomy alone is evident. This is particularly clear in the setting of paravalvular involvement or in patients with prosthetic valves and implanted cardiac devices. Important diagnostic and prognostic information can also be garnered from the use of CT chest and nuclear imaging modalities such as PET/CT to identify extracardiac lesions such as those adhering to extracardiac components of devices or peripheral emboli.

Future advances including higher resolution imaging, further reduction of radiation exposure within CT protocols, and more bacteria specific radiolabeling will only add to the power and utility of multimodality imaging. Each modality has its specific strengths and limitations and there is, as yet, no agreement on standard imaging protocols for multimodality imaging in endocarditis. Given the strengths of specific imaging for specific types and sequelae of infective endocarditis, authors who have proposed algorithms have tended to separate these into streams, for example, separate algorithms for native valve endocarditis, and endocarditis related to prosthetic valves or cardiac devices.

The literature on any pathology is arguably only as valuable as its real-world application and what is been made clear here is the yield of each imaging technique is operator experience dependent and reliant on the use of standardised protocols. Some imaging modalities lend themselves better to this standardisation. For example, CT imaging including PET/CT might be more uniformly obtained in shorter imaging sequences than, for example, longer SPECT CT or MRI protocols. Similarly, these imaging techniques might all yield more uniform results than echocardiography where imaging is perhaps most significantly altered by body habitus, the patient’s tolerance of the procedure, and the manual dexterity of the operator. Furthermore, many imaging modalities may be limited by individual institutions’ resources and experience. This makes work on developing higher resolution imaging techniques even more vital as even lower resource settings, reliant on echocardiography alone, may benefit from improvement of echocardiography techniques with higher spatial, temporal, and three-dimensional resolution.

Many of the limitations and special considerations detailed here for a CHD population also require assessment in a centre with a good understanding of this patient population and their imaging findings both pre- and post-surgical repair. Furthermore, much of the literature considered here concerned studies with very heterogenous populations (some even mixing adult and paediatric patients) and did not separate their CHD population from non-CHD, even if this was considered within discussion of the results. The majority of studies in this group of patients are cohort studies, with the best evidence to date obtained from prospective, multi-centre trials in relatively large cohorts [3]. The need for more well-powered studies is clear, particularly studies addressing a specific CHD cohort. It would also be of great value to consider specific paediatric CHD populations. This group of patients consists of quite different underlying cardiac substrates and sometimes presents more of a challenge in identifying the aetiology of febrile illness amongst common childhood community-acquired infections. They are also a group in whom consideration of the demands of specific imaging modalities is vital. For example, they may not be able to tolerate echocardiography, or a long MRI protocol and careful consideration has to be given to the risks and benefits of sedation for the purposes of imaging particularly in those with underlying CHD.

## 8. Conclusions

This review has summarised some the main attributes and limitations associated with the use of non-invasive imaging techniques in the diagnosis, prognostication, and treatment of infective endocarditis with particular attention paid to a congenital heart disease population. It has included echocardiography, cardiac MRI, cardiac CT, and nuclear medicine. Diagnosis of infective endocarditis remains challenging in a number of patients who do not fit the Duke Criteria for a definite diagnosis of IE. This cohort of patients is only likely to grow larger in future as medical advances mean more children are surviving for longer with CHD and the use of prosthetic vales and implantable cardiac devices becomes more widespread. Imaging continues to play a vital role in diagnosis and decisions around further management and to utilise this to its full potential the need for further research on the use of multimodality imaging in IE is clear particularly in the field of paediatric and adult congenital heart disease. Furthermore, an agreement on standardised adjunctive imaging protocols will not only help to support their use but also allow for more reproducible studies. All of this with the ultimate aim of making early and accurate diagnosis of IE in patients thus minimising unnecessary hospital stays and surgical interventions for patients with CHD which carry with them a clear physical and psychological burden for the patient and their family.

## Data Availability

Not applicable.

## Abbreviations

2D	Two Dimensional
3D	Three Dimensional
4D	Four Dimensional
bSSFP	Balanced Steady-State Free Precession
ACHD	Adult Congenital Heart Disease
CAD	Coronary Artery Disease
CCT	Cardiac computed tomography
CHD	Congenital heart disease
CMR	Cardiovascular Magnetic Resonance
CT	Computed tomography
ECV	Extracellular Volume
EGE	Early Gadolinium Enhancement
ESC	European Society of Cardiology
GBCA	Gadolinium-Based Contrast Agent
GLS	Global Longitudinal Strain
HF	Heart Failure
IE	Infective endocarditis
LA	Left Atrium
LGE	Late Gadolinium Enhancement
LoE	Level of Evidence
LV	Left Ventricle
LVEDD	Left Ventricle End-Diastolic Diameter
LVEF	Left Ventricle Ejection Fraction
LVH	Left Ventricle Hypertrophy
LVM	Left Ventricle Mass
MRI	Magnetic Resonance Imaging
NSF	Nephrogenic Systemic Fibrosis
PC	Photon counting
PCI	Phase Contrast Image
ROI	Region of interest
RV	Right Ventricle
SAX	Short Axis
STE	Speckle Tracking Echocardiography
TDI	Tissue Doppler Imaging
TOE	Transoesophageal Echocardiography
TTE	trans-thoracic echocardiography
TR	Tricuspid Regurgitation
